# Novel variants in *POLH* and *TREM2* genes associated with a complex phenotype of xeroderma pigmentosum variant type and early‐onset dementia

**DOI:** 10.1002/mgg3.1491

**Published:** 2020-09-16

**Authors:** Izadora Fonseca Zaiden Soares, Denise Maria Christofolini, Lis Gomes Silva, David Feder, Alzira Alves de Siqueira Carvalho

**Affiliations:** ^1^ Department of Neurosciences Centro Universitário Saúde ABC Santo Andre São Paulo Brazil; ^2^ Department of Collective health Centro Universitário Saúde ABC Santo Andre São Paulo Brazil

## Abstract

**Background:**

Xeroderma pigmentosum (XP) is a rare, genetically heterogeneous, autosomal recessive disorder caused by defects in the genes involved in repairing DNA damaged by ultraviolet radiation. These defects lead to a propensity to develop skin cancer at early ages as a hallmark, and progressive neurological degeneration can be observed in around 25% of patients. Eight clinically heterogeneous groups have been identified so far (XPA to XPG and XPV). Xeroderma pigmentosum variant type (XPV) is associated with pathogenic variants in *POLH* on chromosome 6, and no neurological dysfunction has been seen in these cases. However, on the same chromosome, it has been shown that *TREM2* is associated with some types of dementia, particularly in patients with a behavioral variant frontotemporal phenotype.

**Methods:**

Gene mutational analysis was performed by whole‐exome sequencing.

**Results:**

We report a case of a Caucasian woman with XP that developed behavioral and cognitive impairment at age 37. Whole‐exome sequencing identified novel homozygous variants in *POLH* c.638C>G (p.Ser213*) and *TREM2* c.154C>T (p.Arg52Cys), classifying the patient as XPV and suggesting that her frontotemporal dementia phenotype could be related to the variant in *TREM2*.

**Conclusion:**

This paper describes a rare case of a patient with two novel variants in the same chromosome associated with XPV and early‐onset dementia.

## Graphical abstract

We report a case of a patient with xeroderma pigmentosum variant type who developed early‐onset dementia, in which two novel gene variants in POLH and TREM2 were found.

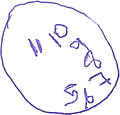

## 1 INTRODUCTION

By the end of the human genome sequencing in 2003, it was possible to analyse the content of chromosome 6, which comprises around 6% of the genome (Mungall et al., 2003). Chromosome 6 has an important role in the innate and adaptive immune system since it contains the genes of the major histocompatibility complex (MHC) (6p21.3) (Mungall et al., 2003). The importance of chromosome 6 goes beyond the role of MHC since several genetically complex diseases have been associated with it, such as dementia and genodermatoses (Chedraoui, Kibbi, & Kurban, 2016; Mungall et al., 2003).

The *POLH* gene (DNA polymerase eta, OMIM: 603968), also located on chromosome 6 (6p21.1), comprises 11 exons and encodes an important DNA polymerase (Pol ɳ), which is able to replicate past noncoding structures by a translesion DNA synthesis (TLS) mechanism (Fassihi, 2013; Yuasa, Masutani, Eki, & Hanaoka, 2000). Pathogenic variants in this gene are related to xeroderma pigmentosum variant type (XPV) disease (Digiovanna & Kraemer, 2012; Fassihi, 2013; Yuasa et al., 2000). Xeroderma pigmentosum (XP) is a rare autosomal recessive disorder caused by changes in the DNA repair pathway related to variants in eight genes, and it is clinically recognized as XPA, XPB, XPC, XPD, XPE, XPF, XPG, and XPV (Digiovanna & Kraemer, 2012; Fassihi, 2013; Gruener & Morley, 2018). It is characterized by photosensitivity and an increased incidence of ultraviolet (UV)‐induced skin lesions and mucous membrane cancers at sun‐exposed sites (Fassihi, 2013). Around 25% of patients with XP can present with progressive neurological degeneration, while XPV patients, representing 20% of XP cases, do not show neurological dysfunction (Digiovanna & Kraemer, 2012; Fassihi, 2013).

In addition, *TREM2* (triggering receptor expressed on myeloid cells 2; OMIM:605086) is another gene located on chromosome 6 (6p21.1) (Yaghmoor, 2014). It is composed of 5 exons and encodes the protein TREM2, an innate immune receptor expressed on the cell surface of activated macrophages, osteoclasts, immature dendritic cells, and microglia (Bianchin et al., 2004). *TREM2* sequence variants are the genetic basis for an autosomal recessive disorder called polycystic lipomembranous osteodysplasia with sclerosing leukoencephalopathy (PLOSL), also known as Nasu‐Hakola disease (Bianchin et al., 2004; Guerreiro et al., 2013). Its hallmarks are multifocal bone cysts, causing a predisposition to fracture during the second or third decade of life, and a behavioral variant frontotemporal dementia syndrome (bvFTD) in the fourth decade of life (Bianchin et al., 2004; Guerreiro et al., 2013).

We report two novel variants on chromosome 6 genes *POLH* and *TREM2* in the same patient, associated with a phenotype of XPV and early‐onset dementia.

## 2 METHODS

We performed a literature review on XPV, *POLH*, *TREM2* and dementia using the Pubmed database and data from OMIM®, Mendelian Heritage Online in Men. The study was approved by the Research Ethics Committee of the Centro Universitário Saúde ABC. Informed consent was obtained to perform the genetic diagnostic assay and for publication from the patient's family. About 5 ml of blood sample was collected from the patient and dissolved in heparin. Genomic DNA was extracted for whole exome sequencing (WES). The GRCh38 version of the human genome was used as a reference (NM_006502.3, NM_018965.4). The ExAC and GnomAD_exome databases were used to search the allele frequency of the *TREM2* and *POLH* variants. Moreover, PROVEAN (http://prove​an.jcvi.org) was used to predict the functional effects of *TREM*2 missense variant. Also, MutPred2 (http://mutpr​ed2.mutdb.org) was used to reason probabilistically about the pathogenicity of amino acid substitutions. Based on homology‐modelling (PDB ID: 3cm9_J), ELASPIC (http://elasp​ic.kimlab.org) was used to predict the structure of wild‐type and variant TREM2 protein (R52C). We also used RCSB (https://www.rcsb.org/struc​ture) in order to know TREM protein domains.

## 3 RESULTS

A 39‐year‐old Caucasian women born in Brazil as the daughter of consanguineous parents (with no similar family symptoms) presented at our Cognition Outpatient Clinic with a history of behavioral and personality changes (according to her mother) at age 37. She was clinically diagnosed with XP at age 7 and had multiple skin tumours.

A period of learning difficulty at school was observed between 6 and 8 years of age, which the patient overcame with specialized assistance. She had normal development, completed high school, graduated as a nursing technician, used to work as a hairdresser, and lived alone up to 36 years of age. At age 37, she was easily distractible and unable to plan and organize her activities. Socially inappropriate behaviors and an interest in religious subjects were present. Six months later, she developed seizures, along with a tendency to take objects to the mouth. In addition, she presented carbohydrate cravings, memory loss, insomnia, and urinary and faecal incontinence. Her functionality decreased significantly, causing her to need assistance for even basic self‐care activities.

**FIGURE 1:**
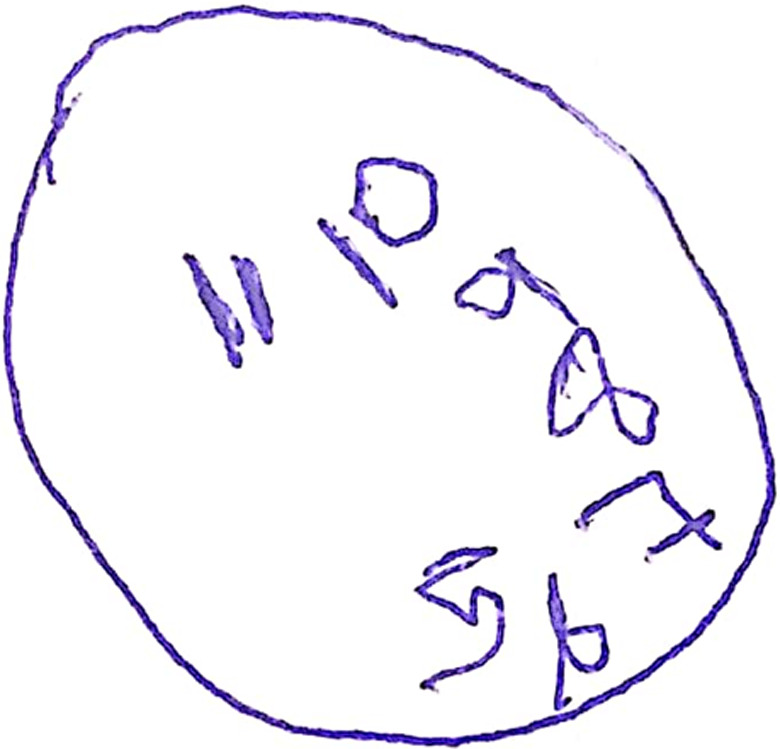
Patient's Clock Drawing Test (CDT)

At first consultation, we observed inattention, disorganization, impulsivity, disinhibition, hyperorality, neglect of personal hygiene, and lack of insight. Neurological examination was unremarkable, except for slight signs of parkinsonism composed of bradykinesia, stiffness, and a parkinsonian gait. Cognitive testing showed executive dysfunction and memory impairment (Mini‐Mental State Examination [MMSE] score of 15/30; Montreal Cognitive Assessment [MOCA] score of 5/30; abnormal Clock Drawing Test [CDT] [Figure 1]; and reduced semantic (score of 3/13) and phonemic (score of 3/13) verbal fluency.

Routine laboratory tests were normal. Magnetic resonance imaging (MRI) of the brain showed diffuse brain atrophy with compensatory ventricular enlargement. A skeletal survey did not show abnormalities. WES was performed and revealed the presence of two novel variants involving the *POLH* gene c.638C>G (p.Ser213*) and the *TREM2* gene c.154C>T (p.Arg52Cys), both observed in homozygosis. The variant R52C was observed twice at ExAC, in heterozygosis. This variant was predicted to be deleterious by Provean (score: −6.615). R52 is located at extracellular domain of TREM2 in the Ig‐like V type portion and the variant C52, in a helical calmodulin binding motif, and the variant was predicted to affect metal binding (probability: 0.70; p‐value:0.0015).

The patient received treatment with antiepileptic drugs, a selective serotonin reuptake inhibitor, and an antipsychotic.

## 4 DISCUSSION

Our proband had a previous clinical diagnosis of XP, which, after WES, was classified as XPV since the variant c.638C>G was identified in homozygosis in the *POLH* gene. This variant promotes the replacement of the serine amino acid at position 213 with a premature stop codon (p.Ser213*). It is absent in about 125,000 individuals in the world population and has not been previously described in the literature. Its molecular mechanism, which involves early interruption of protein translation, combined with the characteristics of the region where it is located and the correlation of this gene with clinical symptoms, indicate that this variant is pathogenic.

XPV cells are deficient in an error‐prone Pol ɳ involved in the DNA repair mechanism of TLS, while other XPs are involved in the repair of UV‐induced photoproducts in DNA by the process of nucleotide excision repair, which is made up of the following two pathways: global genome repair (GGR) and transcription‐coupled repair (TCR) (Fassihi, 2013; Yuasa et al., 2000).

The nature of the damage to the central nervous system is still unknown, but it seems to be associated with the TCR pathway, which is functional in XPV (Digiovanna & Kraemer, 2012; Fassihi, 2013; Gruener & Morley, 2018). Munford et al. (2017) studied one of the densest known areas of XP patients in the word, an isolated community known as the “Moon people,” with a high frequency of consanguineous marriages in central western Brazil. He found a genetic cluster of two pathogenic variants in the *POLH* gene (c.764+1 G>A and c.907 C>T) in 17 patients among the approximately 1000 inhabitants. None of these XPV patients presented developmental problems or neurological dysfunction. However, the article does not describe in detail how this neurological assessment was executed.

According to our literature review, previous reports on the molecular defects in XPV patients described more than 60 variants in *POLH* gene, and there has been no description of neurological manifestations among them, independent of the type of mutation and of the domain of the gene that the variant is located (Broughton et al., 2002; Hong et al., 2018). In addition, we did not find any study that has carried out a complete neuropsychological assessment in these patients. Patients with forms of XP that present neurodegeneration usually develop normally until 2 years of age and gradually evolve with progressive cognitive impairment, reaching a median age of death of 29 years, which is younger than XP patients without neurological degeneration, who die around 37 years of age (Anttinen et al., 2008; Fassihi, 2013). This clinical picture differs greatly from our patient's clinical presentation, whose symptoms, mainly personality and behavioral changes, began at 37 years of age.

Even though most neurons in the brain are in a postmitotic phase and do not replicate throughout life, during neurogenesis they do replicate (Lodato et al., 2017). Therefore, we hypothesized that during prenatal neurogenesis the defective Pol ɳ would not synthesize DNA correctly, meaning that at the end of birth there would be mutations in brain cells that, added to the somatic mutations, can accumulate over time, predisposing individuals to develop later neurological dysfunction.

Moreover, surprisingly, our patient's WES showed a novel variant in *TREM2*. Recently, variants of *TREM2* have been reported as significant risk factors for late‐onset Alzheimer's disease (LOAD), early‐onset Alzheimer's disease (EOAD), pure early‐onset dementia, frontotemporal lobe degeneration (FTLD), and FTD‐like syndrome without the presence of bone cysts, differing from PLOSL, which is the classic disease related to pathogenic variants in *TREM2* that involves early‐onset dementia and recurrent bone fractures (Bianchin et al., 2004; Chouery et al., 2008; Giraldo et al., 2013; Guerreiro et al., 2013; Yaghmoor, 2014). The behavioral and cognitive symptoms of our patient are consistent with possible bvFTD, according to the Frontal Temporal Dementia Consortium (Rascovsky et al., 2011). Although the mechanisms of *TREM2* mutations leading to neurodegeneration are unknown, it is interesting to highlight that TREM2 is one of the highest expressed cell surface receptors on microglia, which plays a key role in the immune response in the central nervous system and in the removal of damaged or apoptotic neurons (Giraldo et al., 2013; Yaghmoor, 2014).

Previous studies have described patients from consanguineous families that presented with behavior and personality changes between 20 and 47 years of age, signs of parkinsonism and seizures, and diffuse cerebral atrophy or regional predominant frontotemporal atrophy according to brain MRI. In all patients, homozygous variants in the *TREM2* gene were found, and none had a bone phenotype (Chouery et al., 2008; Giraldo et al., 2013; Guerreiro et al., 2013). These cases are similar to our patient who presented the same symptoms above mentioned, corroborating the hypothesis that the patient's variant may be pathogenic.

Additionally, it is very important to consider the genetic findings in our case. The *TREM2* variant c.154C>T found in this patient was in homozygosis, promoting the replacement of the amino acid arginine at codon 52 (which is moderately conserved in several biological species) by cysteine (p.Arg52Cys). *In silico* tools for the prediction of pathogenicity suggest that this substitution of arginine with cysteine is potentially harmful. This variant is present in heterozygosis in only 6 out of about 125,000 individuals in the world population and has not been previously described in the medical literature. A distinct variant in this same codon, pArg52His, also a missense mutation, has been previously reported as a possible susceptibility allele to the development of LOAD and has not been associated with the biallelic phenotype of PLOSL, so far. Thus, until now, Arg52Cys has been considered a variant of uncertain significance (VUS).

It is also important to note that this variant is located in the extracellular domain of the protein, possibly reducing TREM2 cell surface expression or affecting ligand binding, thus increasing the risk of neurodegeneration and early‐onset dementia. In addition, the change to Cys could still affect overall protein structure, contributing to the possibly pathogenic effect of this variant. These considerations are consistent with other previous works that described variants associated with AD and FTD that reduce the TREM2 surface expression (Sirkis et al., 2016; Song et al., 2017). The investigation of reversible causes of dementia was negative, and no other gene related to dementia was identified.

## 5 CONCLUSION

In conclusion, this paper describes a rare case of a patient with two novel variants in the same chromosome, one in *POLH* that is known to be pathogenic, causing XPV, and the other in *TREM2*, considered VUS until now. However, this *TREM2* variant could perhaps justify the cognitive and behavioral symptoms, considering the similarity among our patient and the other cases of FTD‐like syndromes without bone cysts previously described (Chouery et al., 2008; Giraldo et al., 2013; Guerreiro et al., 2013). Further progress in understanding the neurodegeneration mechanism in XP and studies that include neuropsychological assessment in XPV are necessary.

## CONFLICT OF INTERESTS

The authors declare that there are no conflict of interests.

## AUTHOR CONTRIBUTIONS

Soares provided the clinical data from patient. Christofolini analyzed the genetic data. Soares and Carvalho wrote the manuscript. Feder revised the manuscript. Silva provided case review. All authors read and approved the final manuscript.
